# The Association Between Placental Residual Blood Volume and Two Placental Transfusion Methods After Delivery at Term

**DOI:** 10.3389/fped.2021.768075

**Published:** 2021-11-08

**Authors:** Tai-Ho Hung, Ya-Chun Chuang, Lulu Huang

**Affiliations:** ^1^Department of Obstetrics and Gynecology, Taipei Chang Gung Memorial Hospital, Taipei, Taiwan; ^2^Department of Obstetrics and Gynecology, Keelung Chang Gung Memorial Hospital, Keelung, Taiwan; ^3^Department of Medicine, College of Medicine, Chang Gung University, Taoyuan, Taiwan

**Keywords:** delayed cord clamping, placental transfusion, umbilical cord milking, cesarean delivery, vaginal delivery

## Abstract

**Background:** Despite reports of the beneficial effects, such as increasing hemoglobin level and iron store in the neonatal period, of delayed cord clamping, or umbilical cord milking after delivery in healthy term-born infants, the duration of delayed clamping or rounds of milking in most previous reports were determined arbitrarily and varied widely across different studies.

**Methods:** We prospectively recruited 80 women with normal singleton pregnancies at 38–40 weeks' gestation. Participants were classified according to the mode of delivery and randomly assigned to either collecting blood from the placenta by umbilical cord drainage (CD) or cord milking (CM), with the placenta left in the uterus. The volume of blood collected, the duration of CD, and the number of rounds of CM were recorded.

**Results:** Collected placental residual blood volume positively correlated with birth weight, placental weight, and length of the cord. When 80% of the total placental residual blood volume collected was set as the threshold, more than 80% of women who delivered vaginally reached this level within 60 s of CD or seven repetitions of CM. This amount of blood could be obtained within 120 s of CD or after seven repetitions of CM in more than 80% of women who underwent cesarean delivery.

**Conclusion:** In most women, regardless of birth weight and placental weight, more than 80% of placental residual blood volume could be collected by CD within 60 s after vaginal delivery, 120 s after cesarean delivery, and seven repetitions of CM in both vaginal and cesarean deliveries.

## Introduction

Despite reports of the beneficial effects, such as increasing hemoglobin level and iron store in the neonatal period, of delayed cord clamping (DCC) or umbilical cord milking (UCM) after delivery in healthy term-born infants, the duration of DCC or rounds of UCM in most previous reports were determined arbitrarily and varied widely across different studies (1, 2). The reported duration of DCC ranged from 30 s to 5 min or until the cord stopped pulsating, and UCM ranged from two to five repetitions (3). However, it is clinically difficult to determine the cessation of cord pulsation, particularly in women who underwent cesarean deliveries (CS) before the onset of labor. Furthermore, it is unclear whether a fixed duration of DCC or number of times of UCM performed is suitable for all newborns, as placental residual blood volume is largely influenced by birth weight, placental weight, and maternal ethnicity (4). With DCC, there are additional concerns regarding the potential risk of hypothermia due to prolonged exposure to the cold temperature in the delivery room or operative theater, and the delay in the commencement of neonatal resuscitation when needed (5).

Therefore, the objectives of this study were to investigate (1) the association between blood volume collected from umbilical cord drainage (CD) and the interval between delivery and cessation of spontaneous drainage; and (2) the association between blood volume collected using cord milking (CM) and the number of repetitions of milking with the placenta left in the uterus after vaginal delivery (VD) or elective CS before the onset of labor in women with normal-term pregnancies. Although the results are not directly translatable, we believe that midwives and obstetricians can utilize and consider the results of this study as an aiding factor when determining an optimal duration of DCC or number of repetitions of UCM after delivery.

## Materials and Methods

We recruited all women with a live singleton pregnancy at 38–40 weeks of gestation admitted between May 19 and June 30, 2021, for VD because of labor pain or for elective CS due to previous hysterotomy or fetal malpresentation. Pregnancies complicated by fetal congenital anomalies, growth restriction, and oligohydramnios were excluded. Women with pregnancy complications such as gestational hypertension, preeclampsia, gestational diabetes mellitus (GDM), acute chorioamnionitis, and medical diseases such as chronic hypertension, pre-gestational type 1 or 2 diabetes mellitus, hyper- or hypothyroidism, and autoimmune diseases were also excluded. Furthermore, women with operative VD and abnormal intrapartum fetal heart rate tracing were excluded, as immediate neonatal resuscitation is frequently needed after birth. This study protocol was reviewed and approved by the Institutional Review Board of Chang Gung Memorial Hospital (approval number: 201901045A3), and the study was also registered at ClinicalTrials.gov (identification number: NCT04898868). Informed consent was obtained from each participant who was enrolled.

Participants were classified according to the mode of delivery and randomly assigned to either collecting blood from the placenta by umbilical cord drainage (CD group) or milking (CM group). Standardized CD and CM techniques were used for this study. Immediately after delivery (<15 s), two clamps were placed at four fingerbreadths from the newborn's abdomen. With the placenta left in the uterus, the cord was cut between the clamps and the newborn was brought to a radiant warmer. For women in the CD group, the placental clamp was removed and the amount of blood drained into a measuring glass was recorded every 20 s, until <2 ml of blood per 20-s interval could be collected. For women in the CM group, the obstetrician supported and clinched the cord with his non-dominant hand near its insertion on the placenta (in CS) or near the introitus (in VD), released the clamp, and then used the thumb and index finger of the dominant hand to milk the entire length of the cord toward its end to squeeze blood into a measuring glass. The end of the cord was clinched, the fingers near the cord's insertion site were released so the cord could refill with blood (usually for 3–4 s), and the procedure was repeated until <2 ml of blood could be squeezed from the cord. The number of times the cord was milked and the volume of blood collected were recorded.

In all cases, oxytocin (intramuscular injection of 10 units in VD or intravenous infusion at a rate of 4 milliunits per min in CS) was administered immediately after delivery of the newborns. CD or CM was performed at a level 20 cm below the introitus in women who delivered vaginally, or on the thighs of mother in women who underwent CS.

Continuous variables are presented as mean ± standard deviation or median (range), and categorical variables as number and frequency (%). Comparisons between groups of CD and CM in women with VD or CS were carried out with Fisher exact test, Student's *t*-test, or Mann–Whitney *U*-test, when applicable. Pearson's correlation analysis was used to determine the relationship between the total blood volume collected by either CD or CM and various factors including maternal characteristics, birth weight, placental weight, and the length and diameter of the umbilical cord. *p* < 0.05 was considered statistically significant. Statistical analysis was performed using SPSS version 26 (IBM Corp., Armonk, NY, USA). Serial changes of the percentage of total placental residual blood volume with the duration of CD or number of rounds of CM were analyzed and plotted using Prism 7 for Mac OS X (GraphPad Software, Inc., La Jolla, CA, USA).

## Results

During the study period, 46 women with VD and 44 women with CS were recruited to participate this study. Ten women were excluded later because of an abnormal fetal heart rate tracing during the second stage of labor, suspicion of placental abruption, and GDM. As a result, data from a total of 80 women, including 40 women with VD (19 in CD group and 21 in CM group) and 40 women with CS (18 in CD group and 22 in CM group), were available for analysis.

There were no differences in the maternal characteristics and neonatal outcomes between these four groups of women (Table 1). In women with VD, significantly more blood was collected by CD than CM, while no difference in the amount of blood between these two methods in women who underwent CS was found. Women with CS had a longer median duration of drainage but similar blood volume compared to women with VD. In regard to CM, women with CS had more blood collected than women with VD, despite similar repetitions of milking.

**Table 1:** Maternal characteristics and neonatal outcomes.

	**Vaginal delivery**			**Cesarean delivery**		
	**Drainage (*n* = 19)**	**Milking (*n* = 21)**	***p-*value**	**Drainage (*n* = 18)**	**Milking (*n* = 22)**	***p-*value**
**Age (y)**						
20–34	12 (63%)	12 (60%)	0.84	9 (50%)	8 (36%)	0.39
>34	7 (37%)	8 (40%)	0.84	9 (50%)	14 (64%)	0.39
**Pre-pregnancy body mass index (kg/m** ^**2**^ **)**						
<18.5	2 (11%)	2 (10%)	0.96	2 (11%)	2 (9%)	0.83
18.5–24.9	15 (79%)	17 (85%)	0.62	12 (67%)	12 (55%)	0.44
>24.9	2 (11%)	1 (5%)	0.61	4 (22%)	8 (36%)	0.33
Weight gain during pregnancy (kg)	10.3 ± 2.3	11.4 ± 2.7	0.18	10.1 ± 3.9	10.7 ± 3.0	0.58
Primiparity	12 (63%)	9 (45%)	0.26	8 (44%)	9 (41%)	0.82
Estimated blood loss (mL)	255.3 ± 66.5	260.0 ± 83.7	0.85	531.7 ± 254.7	566.4 ± 331.3	0.72
Hemoglobin before delivery (g/dL)	11.7 ± 1.7	12.6 ± 1.1	0.08	11.6 ± 1.3	12.2 ± 1.2	0.13
Hematocrit before delivery (%)	35.1 ± 3.9	36.9 ± 3.0	0.11	35.1 ± 3.4	36.3 ± 2.9	0.25
Hemoglobin postpartum day 1 (g/dL)	11.6 ± 1.7	12.3 ± 1.1	0.11	10.8 ± 1.3	11.1 ± 1.4	0.55
Hematocrit postpartum day 1 (%)	34.7 ± 5.2	36.9 ± 3.3	0.12	36.7 ± 57.2	32.7 ± 3.7	0.33
Birth weight (g)	3180.5 ± 318.9	3072.0 ± 293.1	0.28	3153.8 ± 395.6	3174.1 ± 354.3	0.87
Placental weight (g)	630.8 ± 119.7	589.0 ± 84.5	0.21	563.2 ± 92.8	582.3 ± 73.7	0.48
Cord length (cm)	40.7 ± 10.0	46.5 ± 10.2	0.09	43.5 ± 13.8	47.2 ± 7.9	0.30
Cord diameter (cm)	1.1 ± 0.2	1.2 ± 0.2	0.41	1.2 ± 0.2	1.1 ± 0.2	0.58
Male infant	9 (47%)	8 (40%)	0.64	7 (39%)	10 (46%)	0.68
Total blood volume (mL)	90.3 ± 30.3	63.4 ± 27.3	0.045	85.2 ± 25.9^a^	86.4 ± 27.1^b^	0.99
Total drainage time (s)	100 (60–120)	–	–	120 (60–180)^c^	–	–
Total number of cord milking	–	8 (6–14)	–	–	9 (6–12)^d^	–

*Data are presented as a number (%), the mean ± standard deviation, or median (range)*.

*p-values are based on Fisher exact test, Student's t-test, or Mann–Whitney U-test*.

a *p = 0.69*;

b *p < 0.01, compared to women undergoing vaginal deliveries with cord drainage and milking, respectively*.

c *p < 0.01*;

d *p = 0.48, compared to women undergoing vaginal deliveries with cord drainage and milking, respectively*.

For women who had undergone VD, birth weight and placental weight significantly correlated with the amount of blood collected by CD and CM. The correlation coefficient was 0.41 (*p* = 0.009) and 0.37 (*p* = 0.02) for birth weight and placental weight, respectively. In addition to birth weight (correlation coefficient = 0.33, *p* = 0.04) and placental weight (correlation coefficient = 0.53, *p* = 0.001), the length of cord also significantly correlated with the amount of blood collected (correlation coefficient = 0.54, *p* < 0.001) in women who had undergone CS.

**Figure 1:**
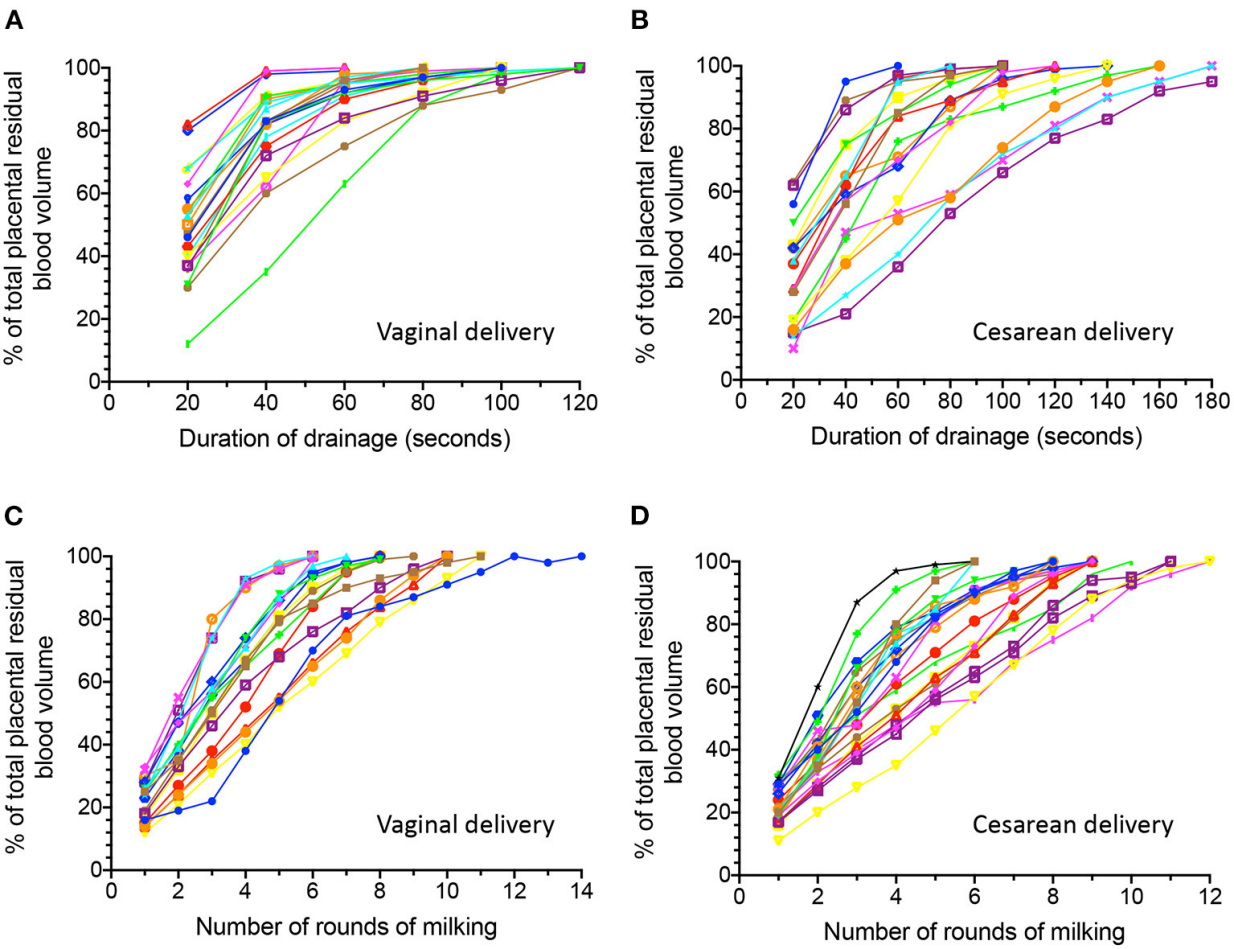
Serial changes in the percentage of total collected placental residual blood volume obtained by spontaneous cord drainage **(A,B)** and milking **(C,D)** after delivery in women with vaginal deliveries and those with cesarean deliveries.

Serial changes in the percentage of total collected placental residual blood volume with CD or CM are presented in Figure 1. When 80% of the total collected placental residual blood volume was set as the threshold, we were able to obtain this amount of blood *via* drainage in 11, 74, and 89% of women after 20, 40, and 60 s, respectively, in women who underwent VD (Figure 1A). The drainage rate was slower in women who had undergone CS; 17, 50, 78, 78, and 94% of these women reached this level of blood volume within 40, 60, 80, 100, and 120 s of drainage, respectively (Figure 1B).

On the other hand, with the same threshold, 24, 62, 76, and 86% of women who had undergone VD reached this level after four, five, six, and seven repetitions of CM, respectively (Figure 1C). Similarly, this amount of blood could be obtained after four, five, six, and seven rounds of CM in 23, 50, 64, and 82% of women who had undergone CS, respectively (Figure 1D).

## Discussion

Similar to previous reports (4, 6), we found that collectable placental residual blood volume correlated with birth weight and placental weight in women who delivered vaginally. The length of cord was an additional factor associated with the amount of residual placental blood in women who underwent CS. Furthermore, we demonstrated that in most women, more than 80% of total collected placental residual blood volume could be obtained by CD within 60 s after VD, 120 s after CS, and seven rounds of CM in both VD and CS, regardless of birth weight and placental weight.

In this study, we found that significantly more blood was collected by CD than CM in women who underwent VD, while there was no difference in the amount of blood between these two methods in women who underwent CS. It is likely that in women with VD, a part of the umbilical cord is within the vagina and therefore cannot be milked, leading to less amount of blood collected than that by CD. On the other hand, the entire length of the cord can be exposed during CS and milking is able to commence from its insertion on the placenta toward the end of the cord, thus making no difference from CD. This also explains that in women assigned to CM groups, those women with CS had more blood collected than women with VD despite similar repetitions of milking.

We also demonstrated that women with CS had a longer median duration of drainage but similar total blood volume compared to women with VD. Furthermore, it took a shorter time for women with VD to reach the same percentage of total collected placental residual blood volume than women with CS. It has been demonstrated that the uterus continues to contract after delivery of the infant. The increasing intrauterine pressure is likely to facilitate the drainage of blood from the placenta. In contrast, women with CS in this study had their operations before the onset of labor. Despite the administration of oxytocin after delivery, the uterus probably contracts less vigorously and requires a longer time to achieve a similar level of blood transfused than that in women with VD.

Our study has several limitations that merit attention. First, we measured collectable residual placental blood volume after delivery without the cord connected to the newborns. This is not exactly compatible with the clinical scenario when DCC or UCM is performed; therefore, the results may not be directly translated to the clinical setting, but instead provides a preliminary theoretical insight. Uterine contractions and gravity have been implied to contribute to the movement of blood from the placenta to the neonate after delivery before the clamping of the cord (7, 8). However, recent experimental or human studies showed that the influence of these factors, if present, is minimal (9–11). In contrast, studies on human neonates have shown that spontaneous breathing during DCC enhances umbilical venous return with a net increase of neonatal blood volume and a reduction of residual placental blood volume (10, 12–14). A Doppler ultrasound study in women with uncomplicated term vaginal deliveries demonstrated that during DCC, venous and arterial umbilical flow occurs and persists with a median duration of or more than 4 min in more than 80% of women after delivery, suggesting that neonatal breathing is a major factor for placental transfusion during DCC (13). Therefore, it is very likely that the measurements of placental residual blood volume with duration of DCC or number of repetitions of UCM would be different from our observations when a neonate with spontaneous breathing and umbilical cord connected to the placenta. Further studies, including measuring hematocrit levels in the umbilical cord and neonates or blood volume in infants with and without DCC or UCM, combined with the results of this study may be the next step in proving more clinically relevant information.

Second, in addition to increased hemoglobin level and iron store in the neonatal period, newborns with DCC have been demonstrated to have a more stable transition from fetal to neonatal circulation compared to those with early cord clamping (10, 15). Although we found that nearly all the residual placental blood was drawn by drainage within 2 min in women with VD and 3 min in women with CS after delivery, these results should not be set as the maximum limit of duration of DCC, as a longer duration of DCC may help maintain cardiac output of the newborns during the first several minutes after birth.

The third limitation of this study is the relatively small sample size, although the patterns of serial changes of the residual placental blood volume collected with CD or CM are quite similar among each participant. Further prospective studies with larger sample sizes are needed to confirm our findings.

In conclusion, our results indicate that in most women, more than 80% of total collectable placental residual blood volume could be obtained by CD within 60 s after VD, 120 s after CS, and seven rounds of CM in both VD and CS, regardless of birth weight and placental weight. These findings are important to midwives and obstetricians for determining the duration of DCC or number of repetitions of UCM to be performed to achieve the maximum benefits of placental transfusion in terms of increasing hemoglobin level and iron store in the neonatal period, while minimizing the risk of cold stress after birth or delaying the commencement of neonatal resuscitation when needed (5).

## Data Availability Statement

The raw data supporting the conclusions of this article will be made available by the authors, without undue reservation.

## Ethics Statement

The studies involving human participants were reviewed and approved by the Institutional Review Board of Chang Gung Memorial Hospital. The patients/participants provided their written informed consent to participate in this study.

## Author Contributions

T-HH conceptualized and designed the study, collected data, carried out the analyses, drafted the initial manuscript, and reviewed and revised the manuscript. Y-CC collected the data, carried out the initial analyses, and reviewed and revised the manuscript. LH reviewed and revised the manuscript. All authors approved the final manuscript as submitted and agreed to be accountable for all aspects of the work.

## Funding

This work was supported by grants from the Chang Gung Memorial Hospital (CMRPG1J0072). The funding body had no role in the study design, in the collection, analysis, and interpretation of data, in the writing of the report, and in the decision to submit the article for publication.

## Conflict of Interest

The authors declare that the research was conducted in the absence of any commercial or financial relationships that could be construed as a potential conflict of interest.

## Publisher's Note

All claims expressed in this article are solely those of the authors and do not necessarily represent those of their affiliated organizations, or those of the publisher, the editors and the reviewers. Any product that may be evaluated in this article, or claim that may be made by its manufacturer, is not guaranteed or endorsed by the publisher.
